# Diagnosis and Management of 1:1 Atrial Flutter in the Setting of Aortic Valve Endocarditis and Embolic Stroke

**DOI:** 10.7759/cureus.8739

**Published:** 2020-06-21

**Authors:** Bisrat Nigussie, Fuad I Abaleka, Maham Suhail, Esmael Yimer, Francesco Rotatori

**Affiliations:** 1 Internal Medicine, Richmond University Medical Center, Staten Island, USA; 2 Cardiovascular Medicine, Richmond University Medical Center, Staten Island, USA

**Keywords:** 1:1 atrial flutter, atrial flutter, endocarditis, septic embolic stroke, cardioversion, aortic valve endocarditis

## Abstract

Atrial flutter is a rapid, regular atrial tachyarrhythmia that occurs most commonly in patients with underlying structural heart disease. Spontaneous 1:1 conduction of atrial flutter is indeed rare, but its diagnosis and management is of critical importance. We describe a case of a 65-year-old man with hypertension, preserved ejection fraction heart failure, end-stage renal disease, Parkinson’s disease, and Alzheimer’s dementia, in whom atrial flutter was associated with 1:1 atrioventricular conduction. Our patient was hemodynamically unstable with aortic valve endocarditis and recent septic embolic stroke. This case report emphasizes the importance of recognition and management to avoid hemodynamic compromise.

## Introduction

Atrial flutter is a rapid, regular atrial tachyarrhythmia that occurs most commonly in patients with underlying structural heart disease [1]. The incidence of typical atrial flutter increases with age, comorbidities, and any disease process that results in secondary atrial dilation [2].

It is rare that endocarditis and atrial fibrillation are found in the same patient [3,4]. Atrial tachyarrhythmias with 1:1 conduction are not common [3,5]. They are usually seen in patients with accessory pathways [5]. Atrial flutter with 1:1 atrioventricular (AV) conduction has classically been described in the setting of class 1A or 1C antiarrhythmic drug therapy [6]. Patients with atrial flutter with 2:1 or higher degree AV block may have received drugs with local anesthetic with or without anticholinergic effect, such as quinidine, procainamide, disopyramide, and flecainide, slowing the atrial rate and allowing 1:1 ventricular conduction [5,7,8]. We present a case of 1:1 conduction of atrial flutter in a hemodynamically unstable patient with aortic valve endocarditis, recent septic embolic stroke, and no history of antiarrhythmic drug therapy.

## Case presentation

A 65-year-old man with hypertension, heart failure with preserved ejection fraction, end-stage renal disease, and Parkinson’s disease was admitted to our hospital for fever, altered mental status, and syncope, which began while the patient was getting dialyzed. On arrival, the patient was tachycardic and hypotensive. The patient was alert and oriented to person and place but not time. He had regular tachycardic rhythm on palpation, a 4/6 diastolic decrescendo murmur best heard in the left third intercostal space, no jugular venous distention, and no pitting edema. The chest examination was normal; the abdomen was soft, non-tender, non-distended, and had positive bowel sounds. Extremities did not show discoloration of nails or rash on the palms or soles of the feet. He was empirically started on vancomycin and piperacillin/tazobactam for possible sepsis with unknown sources and was admitted to the intensive care unit. His home medications (amlodipine, atorvastatin, isosorbide mononitrate, aspirin, pantoprazole, metoprolol, and mirtazapine) were resumed.

The normal reference range of troponin is <0.015 ng/mL; the patient had elevation in troponin, which subsequently trended down as shown (2.720 ng/mL > 2.420 ng/mL > 2.570 ng/mL > 1.3 ng/mL). Thyroid function tests and complete blood count were within normal limits, except for white blood count, which was 10.6 × 10^9^/L. The basic metabolic panel showed an elevation of BUN (blood urea nitrogen) and creatinine, but that is expected given that the patient has an end-stage renal disease.

**Figure 1:**
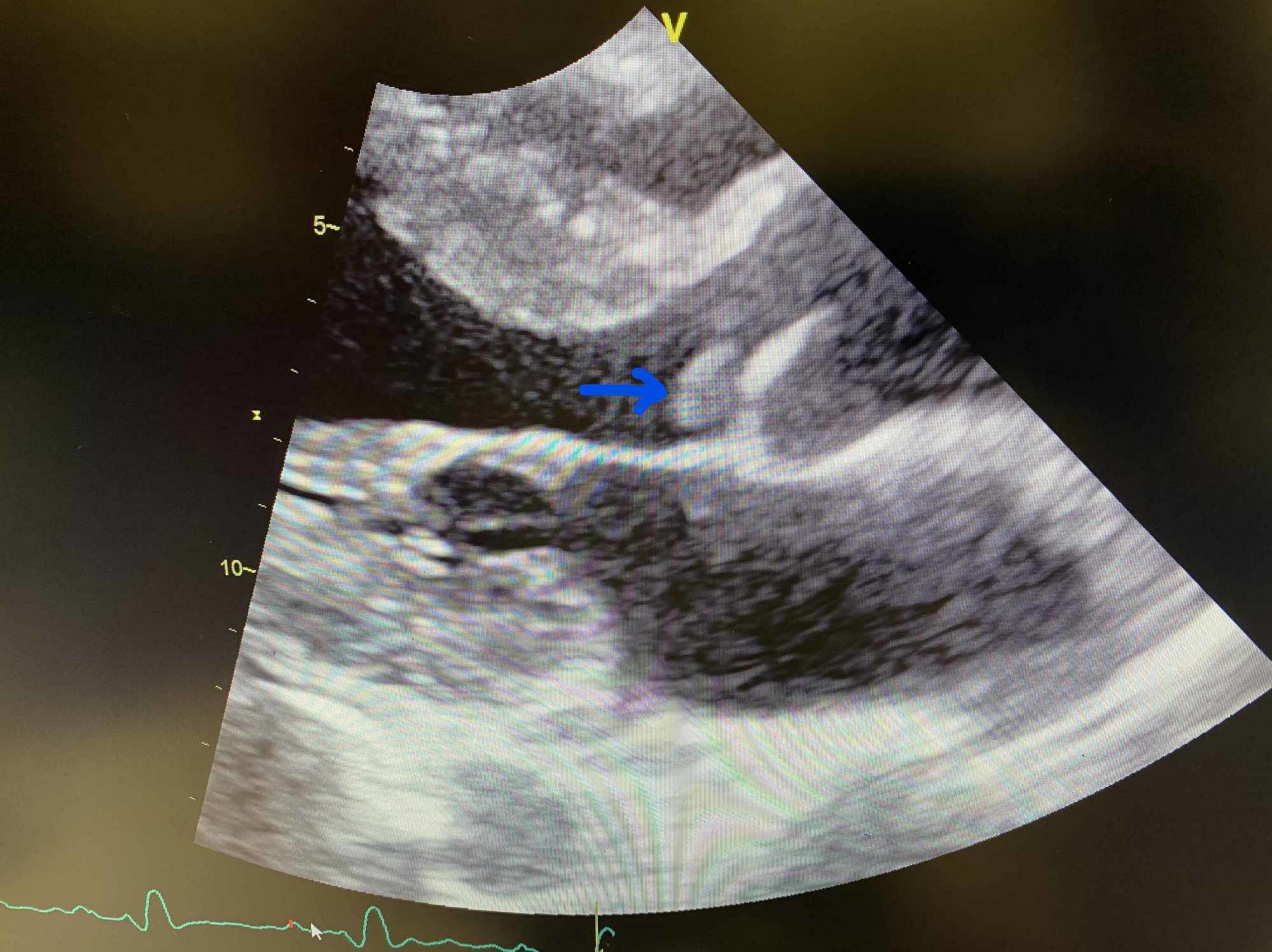
Echocardiogram showing a 1.3 x 0.7 cm aortic valve vegetation

**Figure 2:**
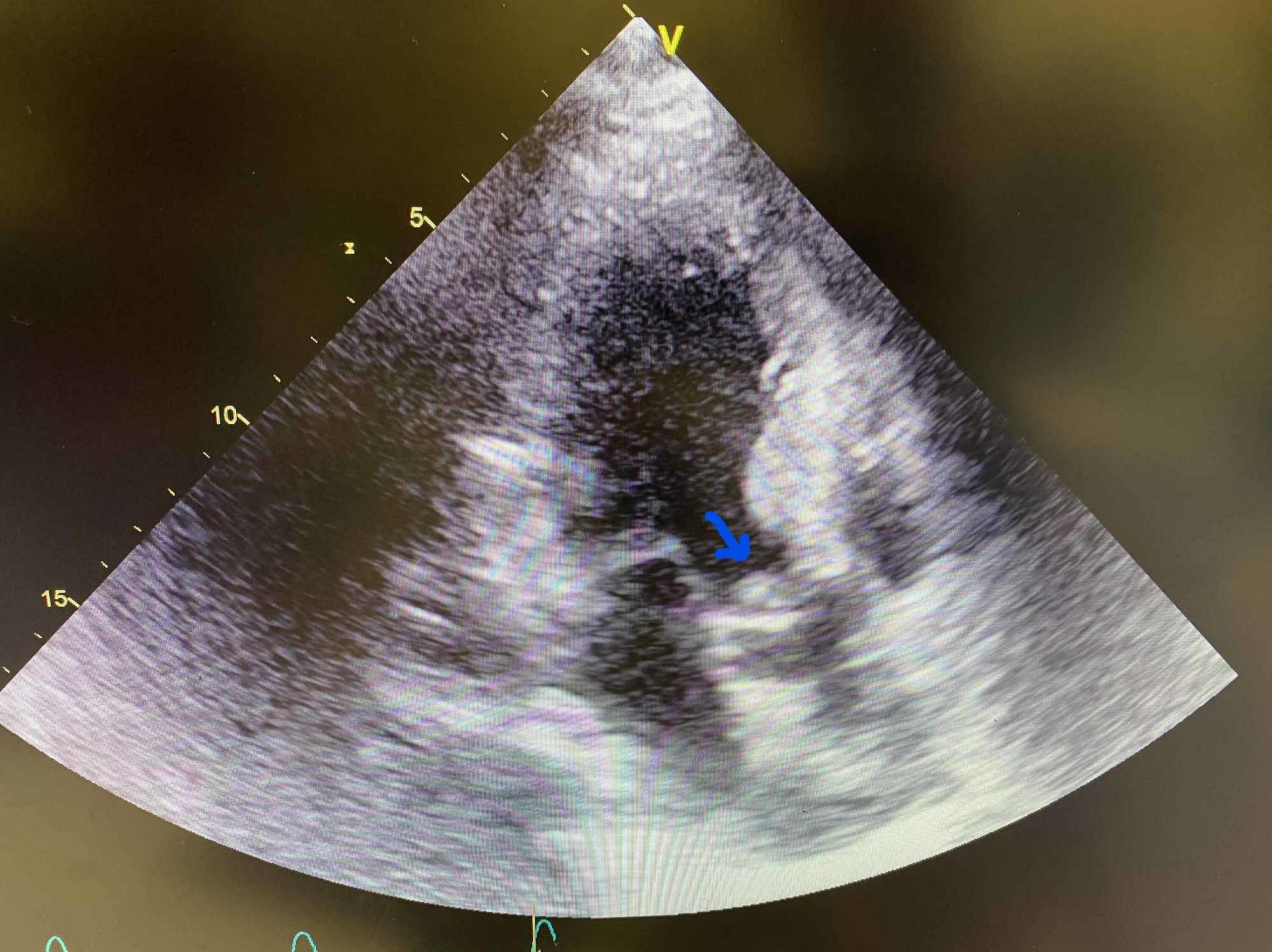
Echocardiogram showing a 1.3 x 0.7 cm aortic valve vegetation

**Figure 3:**
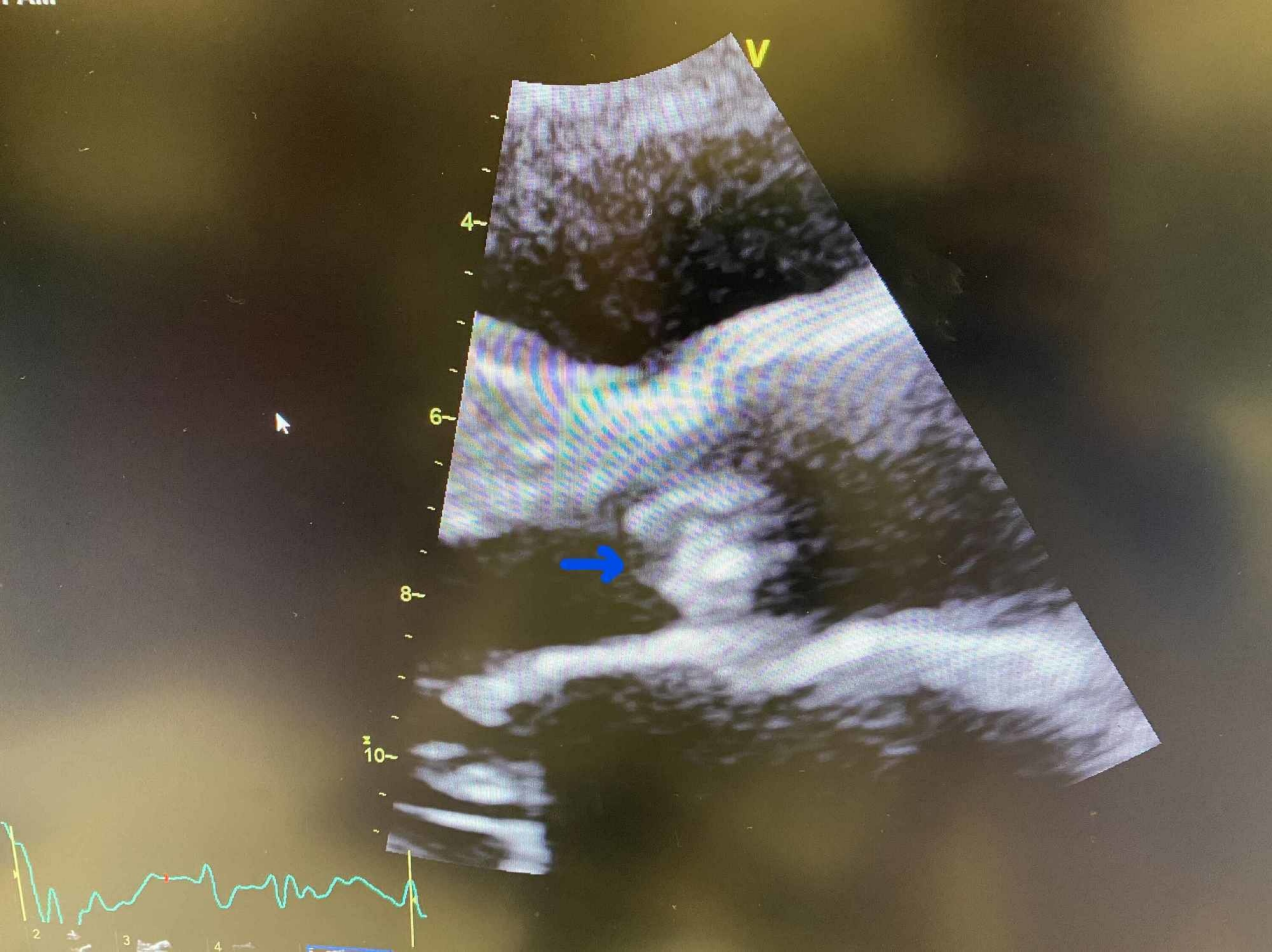
Echocardiogram showing a 1.3 x 0.7 cm aortic valve vegetation

**Figure 4:**
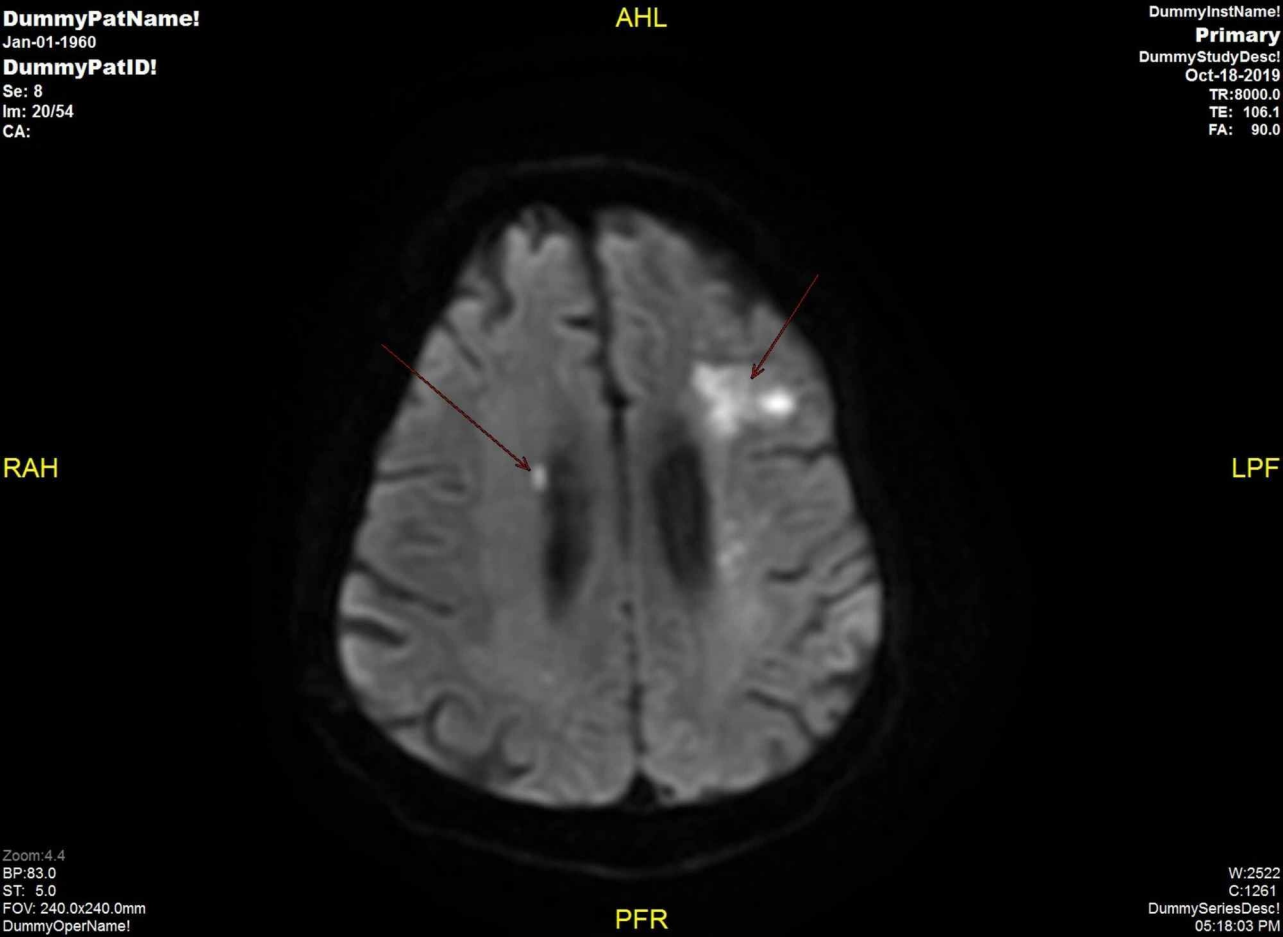
MRI showing embolic stroke

An EKG showed sinus tachycardia with premature atrial complexes, a right bundle branch block (RBBB), and left anterior fascicular block. Transthoracic echocardiography demonstrated aortic valve vegetation with moderately severe aortic regurgitation, severe pulmonary hypertension, and an oval, large mobile density on the left-ventricular side of aortic valve measuring 1.3 x 0.7 cm consistent with endocarditis, as seen in Figures 1-3. CT scan of the head showed marked small vessel disease with an old right frontal infarct. MRI of the head revealed multiple areas of acute/subacute infarctions in multiple vascular territories (left corona radiata bilaterally, right frontal and right temporal lobes, and right parietal, lobe as shown in Figure 4).

**Figure 5:**
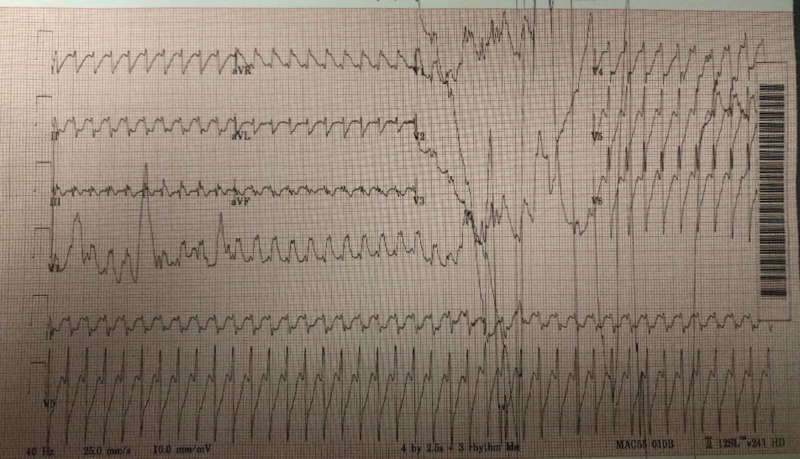
Pre-shock EKG with atrial flutter 1 :1 conduction with a ventricular rate of 242 bpm and RBBB RBBB, right bundle branch block

**Figure 6:**
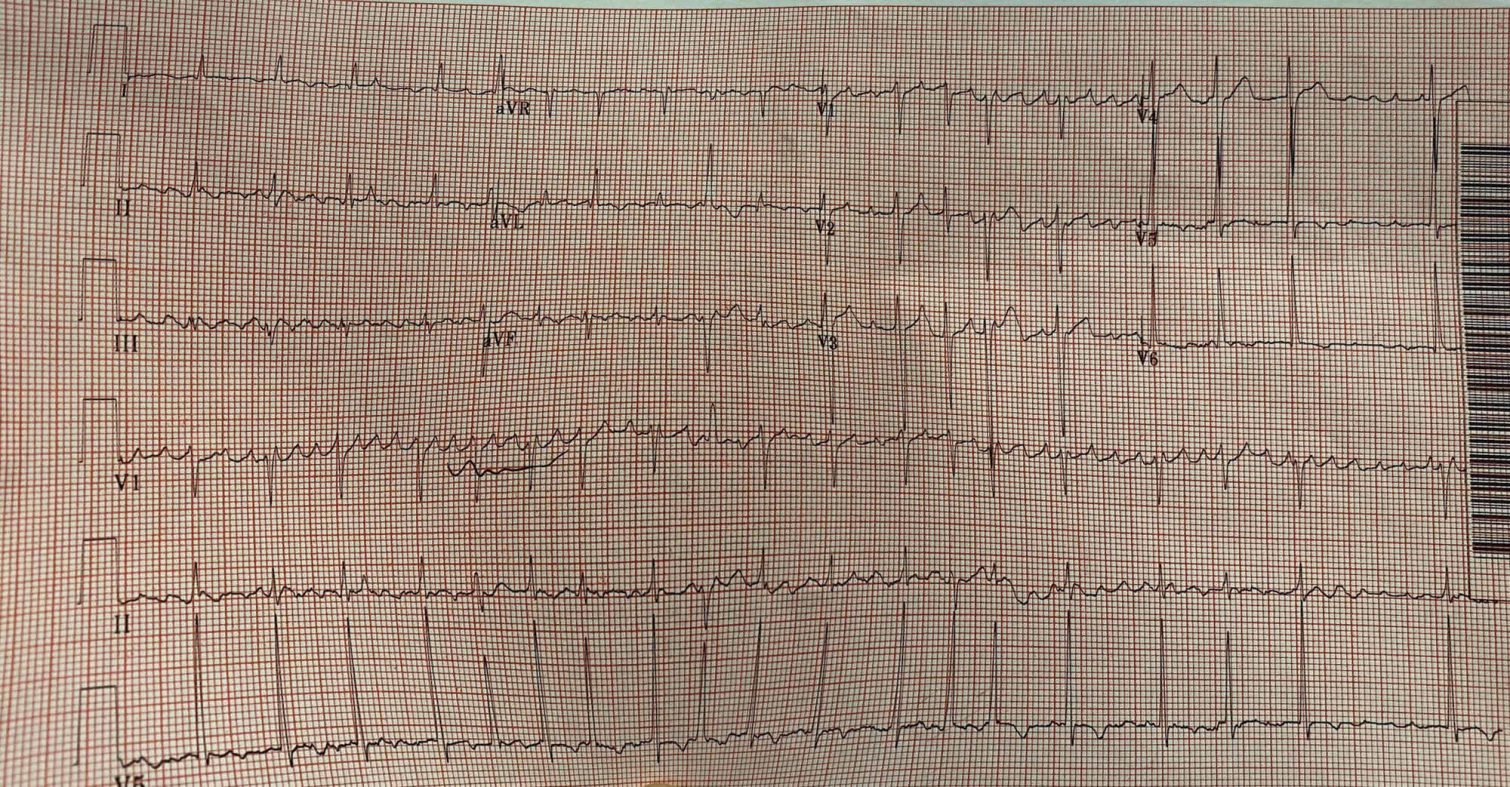
Post-shock EKG showing atrial fibrillation conduction with improved heart rate from 242 to 117 bpm

Blood cultures grew methicillin-resistant *Staphylococcus aureus*, and empiric antibiotics were continued. Two days later, the patient was found to be obtunded, hypotensive, tachycardic, febrile, and tachypneic. EKG showed atrial flutter with a 1:1 conduction with a ventricular rate of 242 bpm and RBBB, as shown in Figure 5. Given the hemodynamic instability in the setting of supraventricular tachycardia, the patient was subsequently electrically cardioverted. The heart rate improved from 242 to 117 bpm with atrial flutter and 2:1 conduction, as shown in Figure 6. The patient was stabilized and transferred to a tertiary center for possible cardiothoracic intervention and further management. In the tertiary center, the patient had an aortic valve replacement and was on a long-term antibiotics treatment.

## Discussion

The second most common atrial tachyarrhythmia is atrial flutter [9-12]. It is an organized reentrant tachyarrhythmia with discernible regular atrial activity usually originating from the cavotricuspid isthmus [13]. When the AV node is functioning normally, not all impulses from the atrium can propagate through to the HIS-Purkinje system; thus, the ventricular rate is lower than the atrial rate during atrial fibrillation and atrial flutter. As a result, it is very rare that an atrial flutter 1:1 AV conduction occurs [14].

It is believed to be 2.5 times more common in men [2]. Although atrial flutter with 1:1 AV conduction is rare, it is important to recognize because it may precipitate rapid hemodynamic compromise [3]. Risk factors for developing atrial flutter include hypertension, heart failure, valvular disease, acute and chronic lung disease, alcohol use, and metabolic disturbances [2]. Symptoms are often related to the rate of ventricular response and may include palpitations, dyspnea, chest pain, presyncope, or syncope.

Antiarrhythmic agents such as flecainide, digitalis, and others have been known to cause a 1:1 atrial flutter [15]. Our patient had 1:1 atrial flutter without taking antiarrhythmic agents. Spontaneous 1:1 conduction of atrial flutter is indeed rare. Capturing a 1:1 atrial flutter outside controlled settings (e.g., electrophysiology [EP] labs) is very rare, and, thus, it is usually underdiagnosed as a possible cause of hemodynamic instability [16,17]. In our patient’s case, it is possible that there was inflammation around the aortic valve area and the edema spread to the surrounding structures such as the AV node, which, in turn, blocked the AV node, allowing the concealed accessory pathways to become the primary pathway to the His-Purkinje system, thus allowing 1:1 conduction. During our literature search, we found one case report that reported Staphylococcus aureus infected endocarditis further complicated by complete heart block due to aortic root abscess [16]. It is not yet clear whether removal of the aortic valve vegetation will reverse the atrial flutter discovered in our case.

In hemodynamically unstable patients with multiple comorbidities, the decision regarding how to proceed with treatment can be challenging due to several factors. In our patient should we proceed with rate control, cardioversion, and/or anticoagulation in the presence of septic emboli and recent ischemic stroke was our dilemma. In patients with hemodynamic instability with atrial flutter, anticoagulation should be started as soon as possible, preferably before cardioversion [18]. However, initiating anticoagulation would put the patient at high risk of hemorrhagic conversion given his recent diagnosis of septic embolic stroke. Furthermore, this patient had aortic septic vegetation, thus performing electrical cardioversion without anticoagulation would increase the risk of another septic embolic stroke. After weighing the risks and benefits, the patient was cardioverted without anticoagulation, and the 1:1 atrial flutter improved to 2:1 atrial flutter. The patient did well post-procedure, and has since remained symptom-free with no new neurological findings up until his time of transfer.

## Conclusions

From this case, we can learn that the diagnosis and management of a 1:1 atrial flutter can be challenging, especially if the patient is hemodynamically unstable and has a cerebrovascular accident due to septic emboli. Spontaneous conduction of 1:1 atrial flutter is a rare occurrence, and it could be difficult to differentiate this arrhythmia from ventricular tachycardia. Common causes of facilitators of a 1:1 conduction need to be ruled out, such as medications, rapid supraventricular tachycardia, and hyperthyroidism. There needs to be more research on how aortic valve endocarditis can cause an AV block and allow conceal pathways to conduct directly from the atrium to the ventricle. Treatment aims to either control ventricular rate or cardiovert to normal sinus rhythm while also anticoagulating. However, there are situations where cardioversion without anticoagulation might be necessary.
